# A Point Mutation in Suppressor of Cytokine Signalling 2 (*Socs2*) Increases the Susceptibility to Inflammation of the Mammary Gland while Associated with Higher Body Weight and Size and Higher Milk Production in a Sheep Model

**DOI:** 10.1371/journal.pgen.1005629

**Published:** 2015-12-11

**Authors:** Rachel Rupp, Pavel Senin, Julien Sarry, Charlotte Allain, Christian Tasca, Laeticia Ligat, David Portes, Florent Woloszyn, Olivier Bouchez, Guillaume Tabouret, Mathieu Lebastard, Cécile Caubet, Gilles Foucras, Gwenola Tosser-Klopp

**Affiliations:** 1 INRA, UMR 1388 Génétique, Physiologie et Systèmes d’Elevage, Castanet-Tolosan, France; 2 Université de Toulouse INPT ENSAT, UMR 1388 Génétique, Physiologie et Systèmes d’Elevage, Castanet-Tolosan, France; 3 Université de Toulouse INPT ENVT, UMR 1388 Génétique, Physiologie et Systèmes d’Elevage, Toulouse, France; 4 INRA, Sigenae, Castanet-Tolosan, France; 5 INRA, UR 0875, Mathématiques et Intelligence Artificielle Toulouse, Castanet-Tolosan, France; 6 Université de Toulouse, Institut National Polytechnique (INP), École Nationale Vétérinaire de Toulouse (ENVT), Unité Mixte de Recherche (UMR) 1225, Interactions Hôtes—Agents Pathogènes (IHAP), Toulouse, France; 7 INRA, UMR1225, Interactions Hôtes—Agents Pathogènes (IHAP), Toulouse, France; 8 INSERM UMR1037, Centre Recherches en Cancérologie de Toulouse, Toulouse, France; 9 Université Toulouse III Paul-Sabatier, Toulouse, France; 10 INRA, UE0321 Domaine de La Fage, Saint Jean et Saint Paul, France; 11 INRA, GeT-PlaGe, Genotoul, Castanet-Tolosan, France; CSIRO, AUSTRALIA

## Abstract

Mastitis is an infectious disease mainly caused by bacteria invading the mammary gland. Genetic control of susceptibility to mastitis has been widely evidenced in dairy ruminants, but the genetic basis and underlying mechanisms are still largely unknown. We describe the discovery, fine mapping and functional characterization of a genetic variant associated with elevated milk leukocytes count, or SCC, as a proxy for mastitis. After implementing genome-wide association studies, we identified a major QTL associated with SCC on ovine chromosome 3. Fine mapping of the region, using full sequencing with 12X coverage in three animals, provided one strong candidate SNP that mapped to the coding sequence of a highly conserved gene, *suppressor of cytokine signalling 2* (*Socs2)*. The frequency of the SNP associated with increased SCC was 21.7% and the *Socs2* genotype explained 12% of the variance of the trait. The point mutation induces the p.R96C substitution in the SH2 functional domain of SOCS2 i.e. the binding site of the protein to various ligands, as well-established for the growth hormone receptor GHR. Using surface plasmon resonance we showed that the p.R96C point mutation completely abrogates SOCS2 binding affinity for the phosphopeptide of GHR. Additionally, the size, weight and milk production in p.R96C homozygote sheep, were significantly increased by 24%, 18%, and 4.4%, respectively, when compared to wild type sheep, supporting the view that the point mutation causes a loss of SOCS2 functional activity. Altogether these results provide strong evidence for a causal mutation controlling SCC in sheep and highlight the major role of SOCS2 as a tradeoff between the host’s inflammatory response to mammary infections, and body growth and milk production, which are all mediated by the JAK/STAT signaling pathway.

## Author Summary

Mastitis is an inflammation of the mammary gland mainly caused by invading bacteria. Ruminants show natural variability in their predisposition to mastitis, and therefore provide unique models for study of the genetics and physiology of host response to bacterial infection. A genome-wide association study was conducted in a dairy sheep population for milk somatic cell counts as a proxy for mastitis. Fine mapping, using whole genome sequencing, led to the identification of a mutation in the Suppressor of Cytokine Signaling 2 gene (socs2). This mutation was shown to cause a loss of functional activity of the SOCS2 protein, which suggested impairment of feedback control of the JAK/STAT signaling pathways in susceptible animals. Additionally, size, weight and milk production were increased in animals carrying the susceptible variant suggesting a pleiotropic effect of the gene on production versus health traits. Results gave strong evidence of the role of SOCS2 in the host’s inflammation of the udder and provided new insights into the key mechanisms underlying the genetic control of mastitis.

## Introduction

Mastitis is an inflammation of the mammary gland mainly caused by bacteria, which develop in the gland cistern after penetration through the teat canal. Mastitis is the main infectious disease of dairy ruminants, with respect to industry and public concern, economic impact, zoonotic potential and animal welfare [1,2]. This disease has occasionally been reported in breast-feeding women [3,4].

How mammals defend against a microbial intra-mammary infection is still poorly understood. Animal models, especially ruminants, have provided useful information about the mechanisms underlying immunity. Mammary defense depends on the early recognition of invading pathogens by sentinels, such as the dendritic cells and macrophages, and also mammary epithelial cells [2]. Early activation leads to the production of soluble factors like cytokines and chemokines that are able to recruit blood cells into the parenchyma and milk to fight the infection by phagocytosis and bacterial killing. This process is finely tuned to avoid collateral damage to the secretory epithelia. Literature data [2,5] corroborate the importance of a rapid influx of neutrophils into the mammary gland, to control the inflammatory process and allow effective and early elimination of the pathogens.

The existence of a genetic basis for mastitis resistance has been well documented in dairy ruminants [6,7,8,9]. Initially, evidence was essentially based on quantifying the polygenic variation of indirect predictors of udder health that could be measured on a large scale in dairy operations. One of the most widely studied predictors was milk somatic cell count (SCC). Indeed, the milk SCC mainly reflects the number of neutrophils that migrate from blood to the mammary gland in response to infection. Measured on a monthly basis, SCC can be interpreted as indicating the consequences of infection and repeatedly high SCC can be associated with the presence of chronic mastitis. In sheep, presence of major pathogens (staphylococci, streptococci, and enterococci organisms) in milk was strongly related to a sharp inflammatory response with increased log SCC means when compared to uninfected half udder [10,11]. Further, Albenzio et al. [12] added to the evidence that immune competence of the mammary gland is related to levels of somatic cell and presence of pathogenic bacteria in ewes with subclinical mastitis. Genetic studies, as reviewed in [2], showed that the estimated heritability for SCC ranged from 10 to 20%, indicating that much of the variation is of genetic origin. The trait is also genetically strongly correlated with resistance to mastitis. This has been further demonstrated in a divergent selection experiment in dairy sheep based on extreme values of log-transformed SCC, i.e. SCS [13]. Measurements of the frequency and duration of bacteria in milk showed that Low-SCS and High-SCS ewes have lower and higher rate of intra mammary infections in natural conditions, respectively. Additionally, bacteriological titer after experimental challenge [14] in the two genetic lines (High vs Low- SCS) demonstrated that bacterial clearance is more efficient in the Low-SCS ewes than in the High-SCS ewes. Accordingly, although SCC and resistance to mastitis are not exactly the same trait (genetic correlation is not 1), genes and mechanisms that underlie those traits are partially common.

The availability of genome sequencing data has opened up new fields of investigation that can be applied to domestic ruminant species with already-sequenced genomes [15,16]. Indeed, the development of high-density single nucleotide polymorphism (SNP) arrays and their application in genome-wide association studies has facilitated the identification of regions controlling production and health traits. These approaches have recently led to the localization of several regions of the genome responsible for some of the variability for udder health traits [2]. However, to our knowledge, except for the direct association with the MHC locus, to date only one QTL for mastitis related traits has been fully described. Indeed, Sugimoto et al. [17] showed that a polymorphism of the bovine forebrain embryonic zinc finger-like gene (FEZL), located in the region of a QTL for SCC on bovine chromosome BTA22, was associated with high and low SCC. Susceptible animals displayed lower expression of FEZL and consequently lower expression of a number of cytokines including TNF-alpha and IL-8. This down-regulation of cytokines was mediated by lower SEMA5A expression.

We mapped the genetic loci determining resistance/susceptibility to SCC as a proxy for mastitis by performing a large-scale QTL analysis using an outbred population of dairy sheep. Following discovery of such loci using a 50K SNP chip, we used fine mapping and whole genome sequencing for molecular dissection of a major region identified on ovine chromosome 3. A possible candidate mutation in the *Socs2* gene was validated by implementing i) genotyping and analysis of the mutation in the discovery population, ii) functional analysis of the isoforms of the SOCS2 protein and, iii) phenotyping of animals carrying contrasted genotypes at the *Socs2* gene. Our results provide new insights into the key mechanisms underlying the genetic control of the host’s response to intra mammary infections.

## Results

### Discovery of a highly significant QTL for SCC on OAR3

QTLs associated with SCC were mapped by performing a genome scan (26 autosomes) in 1009 dairy sheep distributed in 33 half-sib families. The SCC trait pertaining to mastitis susceptibility was the lactation average of somatic cell score (LSCS) as this trait is highly correlated with intra mammary infections. The phenotype was the daughter yield deviation provided by the routine genetic evaluations. These represent the average performance of the daughters of a sire, corrected for the environmental effects and the genetic value of the mates.

**Fig 1:**
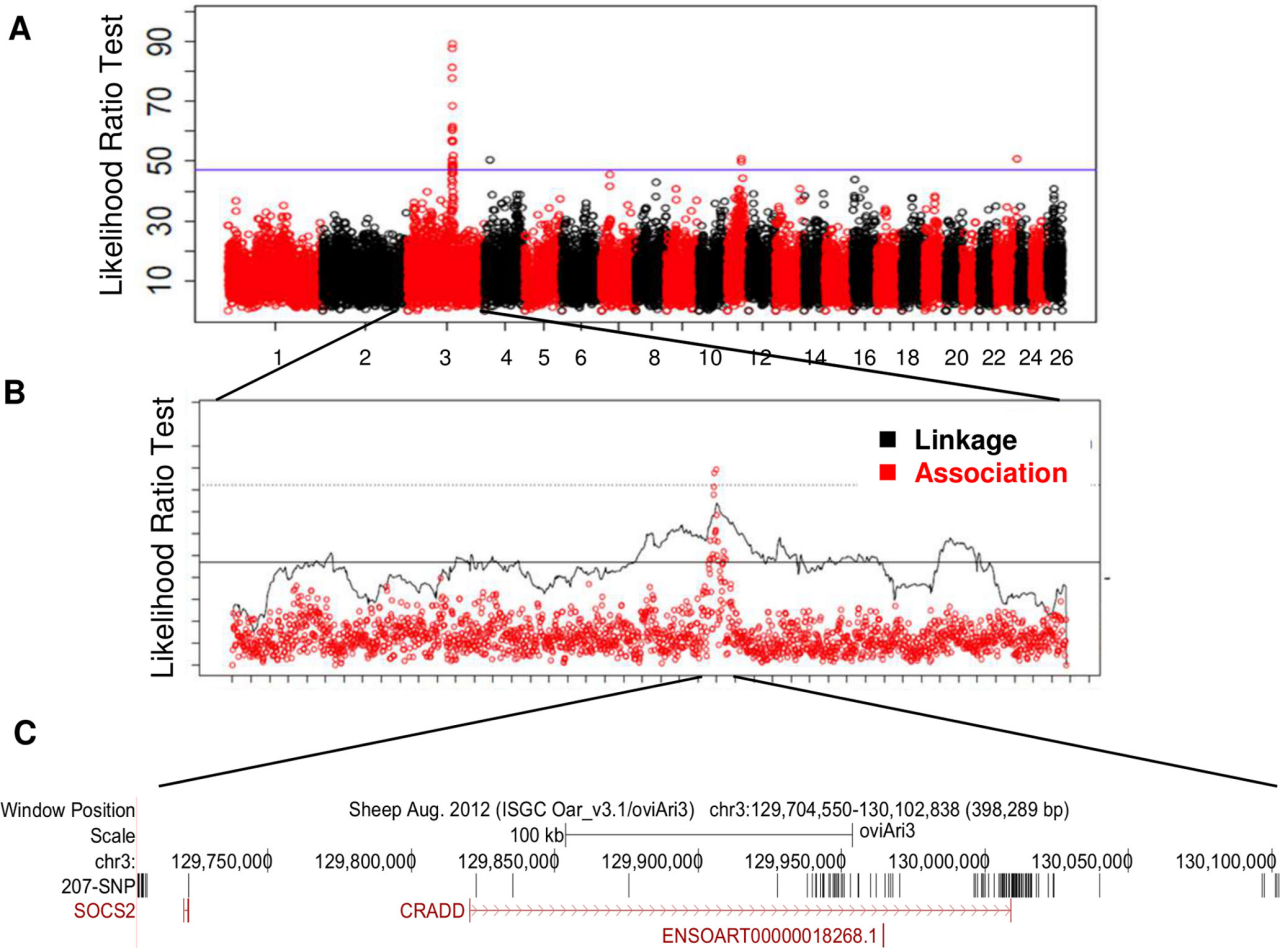
Genome scan for the milk somatic cell count trait LSCS in a grand-daughter design of 1009 dairy sheep identifies a highly significant QTL on chromosome OAR3. **(A)** Manhattan plot for likelihood ratio test profile for LSCS trait based on haplotype-based association analyses on the 26 ovine autosomes. **(B)** Global likelihood ratio test (LRT) profile for LSCS trait on chromosome OAR3 based on both linkage and haplotype-based association analyses. The 5% genome-wide thresholds are indicated for association (solid line) and linkage (dotted line) analyses. (**C**) Localisation of the 207 SNP in the OAR3 QTL confidence interval. SNP were identified using whole genome sequencing in a trio of rams.

All animals were genotyped with the 50K OvineSNP50Beadchip (Illumina, San Diego, CA). Haplotype-based linkage and association analyses were used to detect QTLs on 22 chromosomes at a 5% chromosome-wide threshold (S1 Table). Five regions on chromosomes OAR3, 4, 11, 16 and 23 exceeded the 5% genome-wide threshold. One highly significant QTL on chromosome 3 (OAR3) was similarly located in the two association and linkage analyses (Fig 1A and 1B; S1 Table). For this OAR3-QTL, the association study provided haplotypes of contrasting susceptibility based on four consecutive SNP: ss836339510—ss836339511—ss836339512—ss836339513, spanning a length of 416kb (129,685,397bp—130,103,393 bp).

### Fine-mapping identification of a non-synonymous mutation in the *Socs2* gene

**Fig 2:**
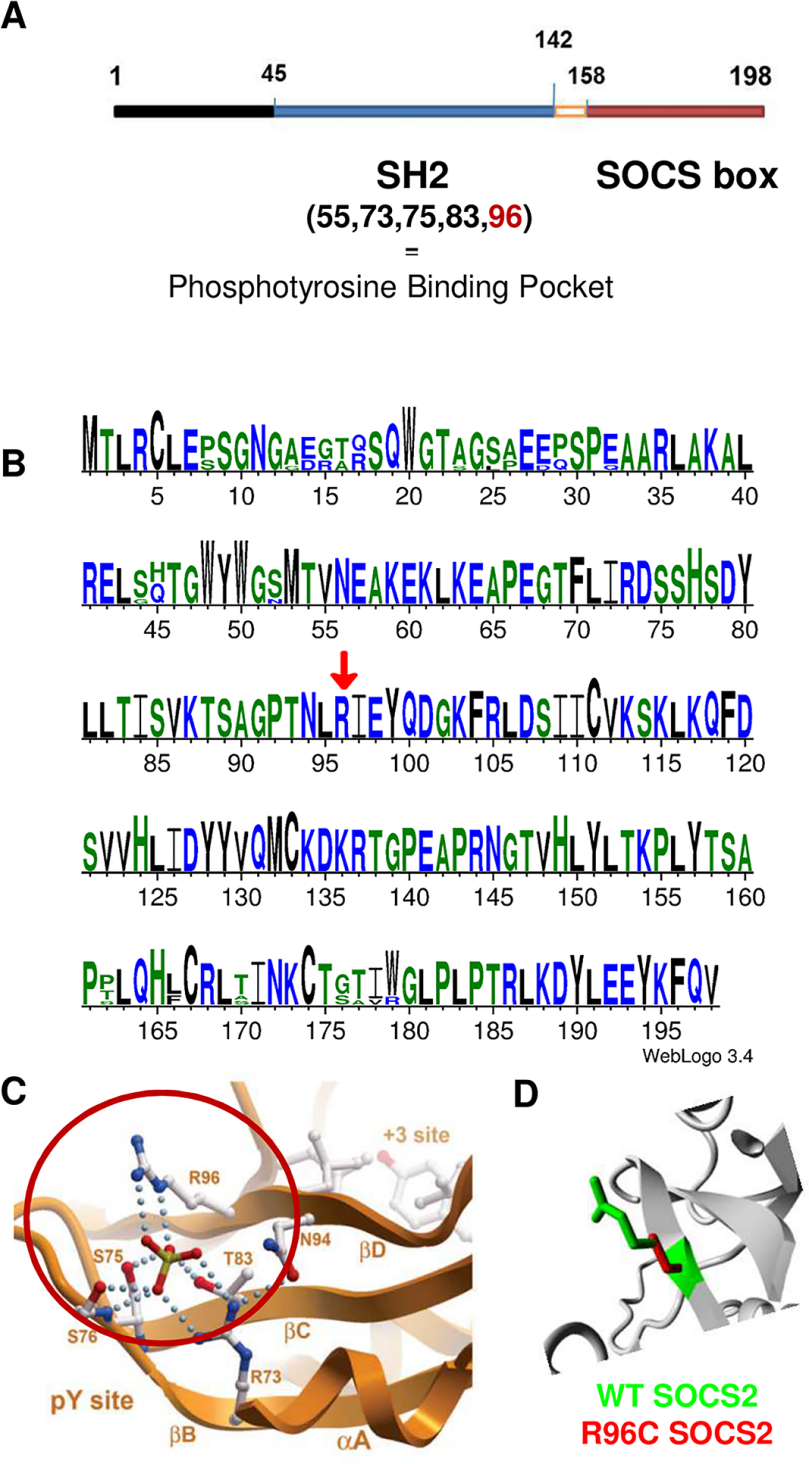
Bioinformatics characterization of *Socs2* and SOCS2 p.R96C mutation. (A) Structure of the *Socs2* gene. (B) SOCS2 is highly conserved across species. The figure was obtained with SOCS2 protein sequences from human, mouse, cattle, pig and sheep species with weblogo software (http://weblogo.threeplusone.com/). Amino-acids are coloured according to their chemical properties. A red arrow shows R96 position. (C) The site of the mutation in the SOCS2 protein structure based on a model predicted by Homotopy Optimization Method, HOPE. **(**D) Close-up of the mutation. The protein is colored grey, the side chains of both the wild-type and the mutant residue are shown in green and red respectively. The mutation is located within the SH2 domain and encodes an arginine (a polar positively charged) to cysteine (mostly hydrophobic) substitution, which can disturb this domain and abolish its function.

The OAR3-QTL was subjected to further fine mapping using whole genome sequencing in a trio of rams (average read-depth of 12X). This trio included a segregating sire carrying the most susceptible haplotype Q (Qq), and two homozygous sons for alternative haplotype alleles (QQ and qq), whose progenies were extremely divergent for the SCC phenotype. Out of the total of 1543 SNPs found in a region of 0.5 Mb, 207 SNPs were retained that were heterozygous (0/1 genotype) for the sire and homozygous, with different genotypes (0/0 or 1/1) for the sons and annotated with the current gtf file for sheep genome (ftp://ftp.ensembl.org/pub/current_gtf/ovis_aries/) (S2 Table). The distribution of the 207 SNP over the OAR3 QTL region is given in Fig 1C. These 207 variations were then submitted to the NCBI databases, dbSNP and dbVar. SNPs were released in the dbSNP Build (B143). Sixty-six SNPs were homozygous for the reference genome base in the susceptible son (0/0), whereas 141 SNPs were homozygous for the alternate base in the susceptible son (1/1). We focused our interest on the variants located in coding or untranslated regions (UTR). Two SNPs were located in the 3’UTR of CRADD gene. Neither of the 3’UTR variants appeared to be conserved, based on GERP scores [18]; the difference between observed and expected GERP scores was -2 for the 130009096 position and -9 for the 130009121 position. Only a single SNP mapped to the coding region of a gene with a non-synonymous change in an amino acid. Modification of the C base in the reference sequence (OAR3, 129722200 bp) to a T in susceptible animals encoded an arginine to cysteine substitution at position 96 (p.R96C) of the Suppressor of Cytokine Signaling 2 (SOCS2) protein. It is noteworthy that this sequence of the SOCS2 protein is highly conserved across species, especially in the region surrounding the mutation (Fig 2A and 2B). The predicted impact of the mutation on the 3D-structure of the protein is shown in Fig 2C and 2D based on a model predicted by Homotopy Optimization Method, HOPE. The mutation is located within the SH2 domain and replaces arginin, a polar positively charged amino-acid by a cystein which is a small and mostly hydrophobic amino-acid. This change can disturb the tri-dimensional structure of the domain and abolish the protein functions.

### The p.R96C SOCS2 mutation abrogates its binding affinity for phosphotyrosine motifs on growth hormone receptor (GHR)

**Fig 3:**
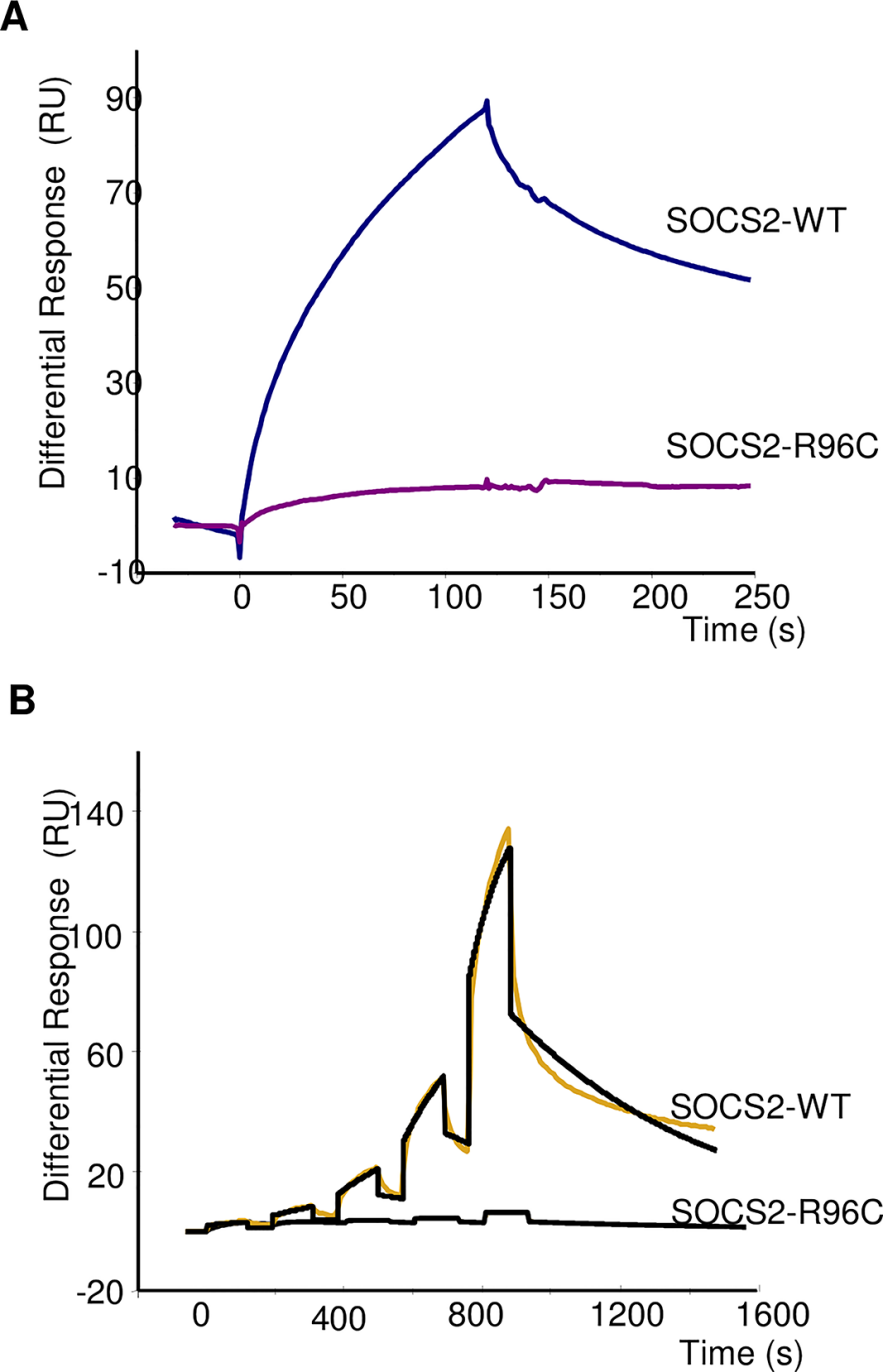
Real-time binding of SOCS2-WT and SOCS2-p.R96C proteins on immobilized pY-GHR. **(A)** Binding analysis was performed on immobilized GHR and scramble pY-peptides (370 RU) at a final concentration of 800 nM. **(B)** Single Cycle Kinetics analysis was performed on immobilized GHR and scramble peptides (75 RU) with five injections of analyte at 100nM, 300nM, 900nM, 2.7μM, and 8.1μM. Analyte injections lasted for 120 s each and were separated by 184-s dissociation phases. The last injection was followed by an extended dissociation period of 10 min. The two sensorgrams recorded for a given analyte were fitted globally to a 1:1 interaction (black curves). Each sensorgram represents a differential response where the reference channel has been subtracted and is expressed in RU as a function of time in seconds.

It has been previously shown that human SOCS2 interacts with endogenous receptors, such as growth hormone (GHR) or erythropoietin (EpoR) receptors, and that this interaction occurs in a phosphorylation-dependent manner at pY595 and pY401, respectively, with the SH2 domain of the protein [19]. We performed biomolecular interactions of recombinant ovine wild type (WT) and p.R96C SOCS2 proteins with phosphorylated peptide from the GHR fixed to a biosensor chip, to compare the rate constants and affinity binding of the two isoforms. When a high concentration of pY-GHR peptide was bound to the support, the observed difference in protein binding was very large (Fig 3A). A similar difference was confirmed after single-cycle kinetics using a wide range of protein concentrations (Fig 3B). Rate constants and affinities for binding of rOvSOCS2 WT on immobilized pY595 GHR were the following: Ka = 8.28 X 10^2^ M^-1^ S^-1^, Kd = 1.6 X 10^−3^ S^-1^, Kt = 3.06 X 10^7^ RU M^-1^ S^-1^ and KD = 2.0 μM. KD were close to those previously published for rhuSOCS2, i.e. 2 vs 1.6 μM [19], an affinity value could not be determined for the p.R96C isoform due to the lack of interaction. These results show that the p.R96C mutation precludes SOCS2 binding to the phosphorylated peptides from at least two endogenous receptors, and is probably associated with a loss of SOCS2 functional activity, suggesting that the p.R96C mutation is associated with a functional knock-out of the *Socs2* gene in T/T homozygous sheep.

### The p.R96C mutation in the *Socs2* gene has a major deleterious effect on LSCS while associated with higher milk production

**Fig 4:**
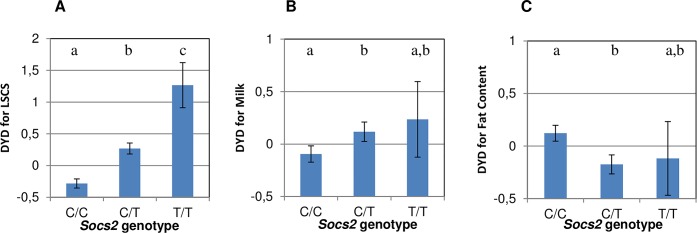
Effect of *Socs2* genotype on milk somatic cell counts (LSCS), Milk Yield and Fat Content in 468 rams. (**A, B and C**) The lsmeans (error bars indicate standard errors) for LSCS (**A**), Milk Yield (**B**) and Fat Content (**C**) from a mixed model including the genotype and sire effect. Traits are expressed as the standard deviation of daughter yield deviations (DYD). The lower case letters (a, b, c) show significant differences in the trait between genotypes. For LSCS, the heterozygotes(C/T) and homozygotes (T/T) were significantly different from wild-type homozygous (C/C) as determined by t-test at p < 0.05. For Milk yield and Fat content, heterozygous (C/T) were significantly different from C/C homozygotes as determined by t-test at p < 0.05.

A PCR genotyping test (KASPar) was implemented to genotype 468 rams in the discovery population for the C/T (p.R96C) mutation in the *Socs2* gene. The frequency of the T mutation, associated with increased inflammation (i.e. elevated SCC), was 21.7%. The frequencies of wild-type (C/C), heterozygous (C/T) and homozygous (T/T) carriers were 58.5%, 40% and 1.5%, respectively. Analysis of variance confirmed that the T mutation involved a dramatic increase in cell counts in the progeny of these rams, especially in homozygous animals, as shown by the average sire breeding values in Fig 4A. Overall, considering the frequency of carrier animals, the *Socs2* genotype explained 12% of the variance of the SCC trait in this population.

To explain the high frequency of the deleterious T mutation in this dairy sheep population, we hypothesized that the *Socs2* genotype might be associated with a beneficial effect on other traits under selection. Indeed, heterozygous C/T rams displayed significantly (p < 0.05) higher progeny milk yields than C/C animals (Fig 4B). Assuming an average milk yield of 280 L and genetic standard deviation of 36.5L (JM Astruc, Institut de l’Elevage, personal communication), the milk increase in homozygous (T/T) carriers is equivalent to + 4.4% when compared to the wild type (C/C).

### The p.R96C mutation in the *Socs2* gene is associated with greater size and weight

**Fig 5:**
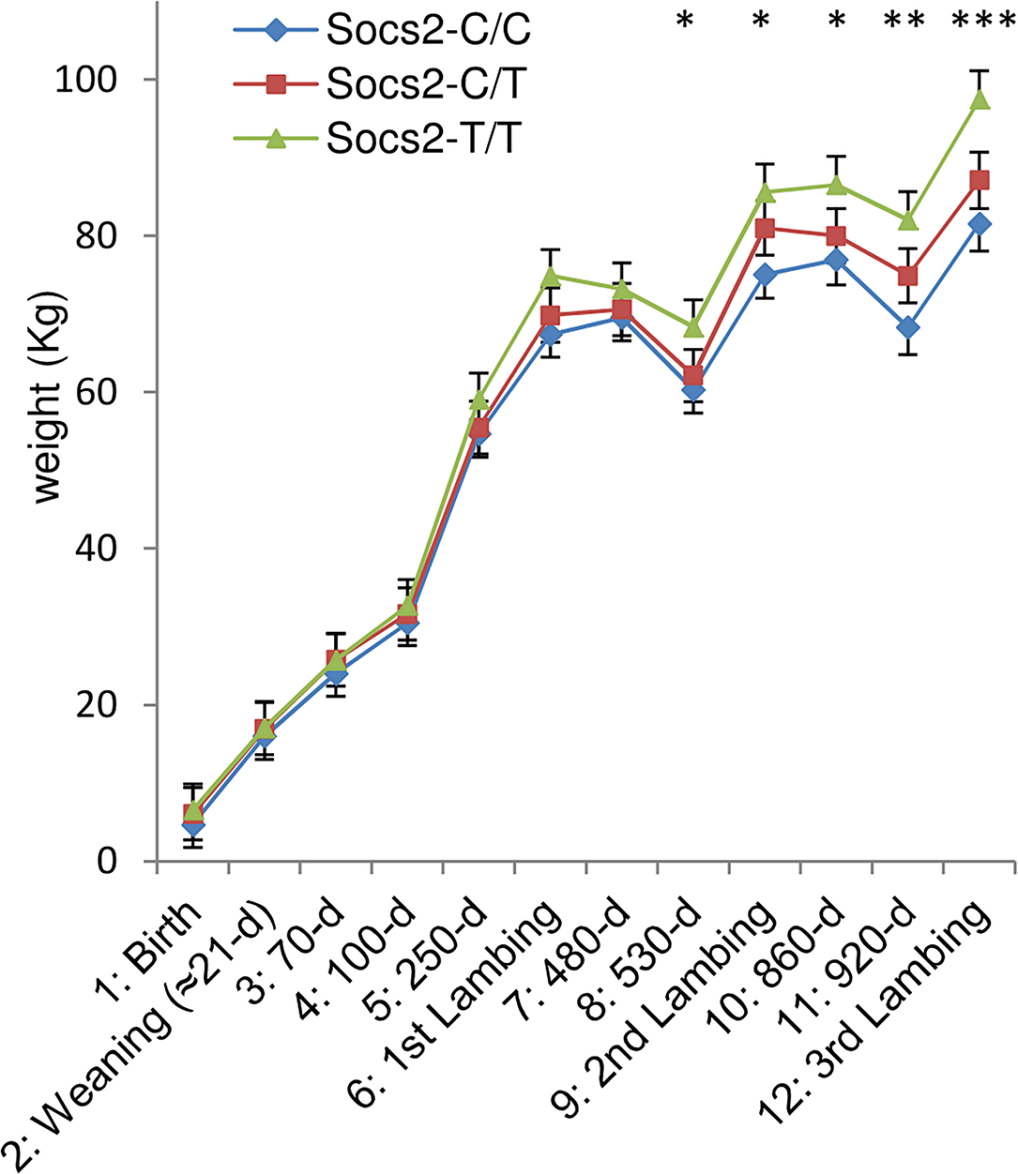
Effect of *Socs2* genotype on body weight in eighteen sheep. The lsmeans (error bars indicate standard errors) for body weight from the mixed model with repeated measures over a 3-year phase including 3 lambing periods. The asterisk shows significant differences in the trait between three genotypes. * For time point 8 to 11, homozygotes (T/T) were significantly different from wild-type homozygous (C/C) as determined by t-test at p < 0.05. ** For time point 11, homozygotes (T/T) were significantly different from wild-type homozygous (C/C) as determined by t-test at p < 0.01. *** For time point 12, homozygotes (T/T) were significantly different from wild-type homozygous (C/C) as determined by t-test at p < 0.001 and heterozygous (C/T) were significantly different from wild-type homozygous (C/C) by t-test at p < 0.05.

**Fig 6:**
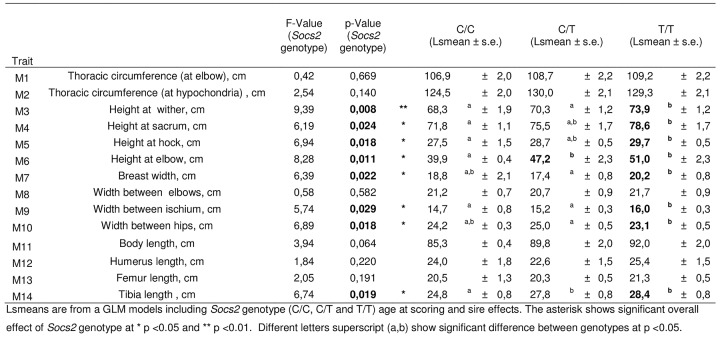
Effect of *Socs2* genotype on body size in eighteen sheep. Lsmeans are from a GLM models including *Socs2* genotype (C/C, C/T and T/T) age at scoring and sire effects. The asterisk shows significant overall effect of *Socs2* genotype at * p <0.05 and ** p <0.01. Different letters superscript (a,b) show a significant difference between genotypes at p <0.05.

It has been previously shown that *Socs2* knock out mice exhibit an unusual gigantism phenotype (Metcalf et al., 2000). We therefore analyzed the growth curve and size of half-sister ewes in relation to their *Socs2* genotype to see if the p.R96C mutation impaired the function of the SOCS2 protein associated with body development. The weights of sheep carrying the T mutation were very much higher than their wild type counter-mates from 17 months of age onwards, i.e., after the end of the first lactation (Fig 5). The average difference between TT and CC was 16Kg at 3 years of age, which was equal to 18% of the average body weight. Similarly, body morphometric measurements showed that 8 out of 14 measures were significantly impacted by the *Socs2* genotype (Fig 6). These included a notable increase in height, width and bone length (tibia) of sheep carrying the T mutation. The average significant difference between TT and CC was 9.2%, and up to 24% for height at elbow. The T mutation in the *Socs2* gene showed an additive effect for both weight and size, the data for heterozygous C/T sheep being mostly intermediate between those of C/C and T/T sheep. These results suggest that SOCS2 is effectively disabled in p.R96C sheep, with regard to well established effects of the gene, and provide a further explanation for the balancing effect of the mutation.

## Discussion

A highly significant QTL for SCC, as a proxy for predisposition to mastitis, was mapped on ovine chromosome 3 (v3: 129,685,397bp—130,103,393 bp). It is interesting to note that among the numerous QTL regions for SCC-based trait reported in the ruminants literature [2] or in the QTL data base (http://www.animalgenome.org/cgi-bin/QTLdb), a dairy cattle study [20] also pointed to the syntenic region on the cattle chromosome BTA5 (23.5–23.9 Mbp) for clinical mastitis occurrence (14.4–26.7 Mbp) and SCS (26.7–30.5 Mbp). Rodriguez-Zas et al. [21] also reported a QTL for SCS on BTA5 (about 7.7 Mbp; http://www.animalgenome.org/cgi-bin/QTLdb). Although the confidence intervals associated with the latter QTLs were large, the present study provides a noteworthy candidate for these QTLs. More generally it appears appealing to systematically screen for mutation in socs2 in various livestock species and ruminants in particular.

We have presented several lines of evidence that the p.R96C mutation of *Socs2* is responsible for this QTL for SCC and explains significant differences in levels of udder inflammation in the studied dairy sheep population. First, by genotyping the mutation in the discovery population, we showed that its frequency was high, i.e., 21.7%, and that the *Socs2* genotype explained up to 12% of the variance of the SCS trait. Second, amongst 207 candidate SNPs, the *Socs2* mutation was the only SNP in the coding region of a gene. This mutation causes an amino acid change in the SH2 domain that is highly conserved across species. Indeed, SOCS2 belongs to a family of 8 proteins: CIS, for cytokine inducible SH2 domain containing protein and SOCS1 to 7 for suppressor of cytokine signaling [22,23]. The proteins in this family share a common structural analogy with a central Src homology 2 (SH2) domain, a C-terminal domain called the SOCS box and a variable N-terminal domain [24,25]. Whereas the C- and N-terminal regions show some variation, the sequence of the SH2 and SOCS box domains of the SOCS proteins is extremely conserved across species, thus suggesting that any non-synonymous sequence variation may have important consequences on the functional role of the protein. Finally, a functional assay for wild type and p.R96C mutated SOCS2 protein showed that the p.R96C mutation prevents SOCS2 binding to phosphorylated peptides from at least two endogenous receptors, and is probably associated with a loss of SOCS2 functional activity. This result suggests that the p.R96C mutation causes with a functional knock-out of the *Socs2* gene.

Other identified SNP were not examined in additional detail after finding the non-synonymous variant in Socs2, supported with functional evidence. Especially, there were two 3’UTR SNPs in the CRADD gene but neither of these variants appear conserved (GERP scores). We cannot exclude, however, that some of those other SNP within the 207 identified may exert a minor role beyond the p.R96C substitution. Genotyping those SNP in independent populations of dairy sheep might help validating the results and refining the relative contribution of each variant to the trait expression.

Many studies have documented the essential role of SOCS proteins (CISH, SOCS1-7) in the regulation of cytokine, growth factors and hormones such as prolactin, growth hormone and erythropoietin [22,23,26]. Proteins In the SOCS-family play roles as negative feedback regulators for those cytokines and growth factors by switching off the Janus Kinase (JAK)/signal transducers and activators of the transcription (STAT) signaling pathway. Gene expression in the nucleus is therefore reduced, resulting in negative control of the cytokine-mediated signal. SOCS1 and SOC3 can directly inactivate the JAK function through a specific region called KIR [22]. The SH2 domain of all SOCS proteins, including SOCS2, binds phosphorylated tyrosine residues in the cytoplasmic tails of cytokine receptors. It is therefore involved in stimulation by ligands and in the downstream competition with STATs. The mutation identified herein, located in the SH2 domain of *Socs2*, most probably prevents activation of SOCS2 by ligands and limits direct or indirect competition with STATs.

The results suggest that a functional knock-out of *Socs2* causes an *in vivo* uncontrolled inflammatory response. This hypothesis is in agreement with the QTL phenotype, i.e., high milk SCC that reflects chronically elevated infiltration of white blood cells in milk of animals carrying the p.R96C mutation in the *Socs2* gene. Because milk SCC are strongly associated with intra mammary infections in sheep [10,11,12] and as both traits are genetically correlated in sheep [14,27], we can hypothesize that elevated SCC associated with the p.R96C mutation may actually be due to more frequent or severe infections, increased mastitis susceptibility and some other immunological dysfunction. However, additional data on health status and immune response between mutant and wildtype animals are needed to confirm this hypothesis. A few other published data corroborate the association of dysfunction of SOCS2 with an impaired immune response. Machado et al. [28] showed that *Socs2* -deficient mice exhibited *in vivo* “uncontrolled production of proinflammatory cytokines “(peritoneal CCL2), “decreased microbial proliferation, aberrant leukocyte infiltration and elevated mortality” upon infection with *Toxoplasma* parasite. In their study, Posselt et al. [29] demonstrated the role of SOCS2 in the counter-regulation and limitation of inflammatory activity of dendritic cells after TLR stimulation. Additionally, SOCS2 appears to be a late-induced SOCS protein [29,30] and has been shown, like SOCS6 and SOCS7, to interact with the SOCS box of other members of the SOCS family, and to be involved in a cross-talk regulation of other SOCS proteins [23,30]. One possible explanation for the role of SOCS2 in immune homeostasis and disease may therefore be the presence of defects in the ability of SOCS2 to regulate SOCS3 expression [30]. Finally, considering the importance of the JAK–STAT signaling pathway in host immunity, Usman et al. [31] investigated single nucleotide polymorphisms in the STAT5A and JAK2 genes in association with mastitis indicator traits (SCC) and some serum cytokines and production traits in Chinese Holstein cattle. They found SNPs in the two genes that were significantly associated with cytokine IL-6, IL-17 and with SCC. Altogether these studies support our finding that genetic variants in the genes involved in the JAK/STAT/SOCS pathway can alter actual functions in this signaling pathway and significantly contribute to the control of inflammatory response to infection. Which biochemical mechanisms, cytokine and associated JAK/STAT molecules are modified by this reported dysfunction of SOCS2, however, remains elusive and needs to be addressed in further studies.

We found a high frequency of the deleterious mutation p.R96C in the population examined and a significant association with higher milk production, growth and size, which raises the question of a balancing selection for increased frequency of p.R96C due to beneficial pleiotropic effects. The large scope of signalling pathways controlled by the SOCS2 protein upholds a pleiotropic effect of the mutation on both immune response and growth and production in the lactating mammal. Indeed, while SOCS2 has been associated with control of immune response and disease outcome in the few above-mentioned studies, SOCS2 is also a well-established negative regulator of GH signalling [22,23,26,32,33] and prolactin signalling [22,23,26]. On the one hand, two models of *Socs2* knockout mice [34] and transgenic mice overexpressing SOCS2 [32] revealed an enlargement of most organs, increased bone growth, weight gain and size in modified mice compared to their wild-type littermates. The results reported by authors, for both models, reinforced the hypothesis that the underlying mechanisms involved suppression of GH signaling by SOCS2 in wild-type animals [32,33,34] mediated by STAT5b [33]. Growth and bone size were both significantly increased in our homozygous p.R96C SOCS2 sheep, substantiating a direct effect of the mutation mediated by the GHR/JAK/STAT signaling pathway. GH is also known to affect mammogenesis and increase milk production in dairy ruminants. Indeed, intra-mammary administration of GH in cattle [35], goat and sheep tends to stimulate milk production during lactation. Zhang et al. [36] showed that udder-size was increased in transgenic goats overexpressing GH in the mammary gland, compared to the controls, suggesting a larger capacity for milk production. Additionally a polymorphism in the GHR has been shown to explain, at least in part, a major QTL for milk production in Holstein-Friesian cattle [37]. In Sarda sheep, significant differences in milk traits were observed among genotypes at polymorphic GHR loci [38]. On the other hand, prolactin (and progesterone) is necessary for the initial development of the alveolar bud of the mammary gland and for further differentiation during secretory initiation and activation, as showed by mouse [39] and cow [40] prolactin/prolactin receptor mutants. Additionally, using gene expression profiling, Harris et al. [41] showed that SOCS2 (and E74-like factor 5) counterbalanced the defects in mammary gland development produced in two prolactin-deficient models, thereby demonstrating the role of SOCS2 in the control of mammary gland development. These results imply a potential direct effect of the p.R96C mutation in the *Socs2* gene on mammary gland development and physiology, and on subsequent milk production, through modified feedback control of GH and the prolactin signaling pathways. More detailed analyses of growth hormone, prolactin and cytokine signaling and SOCS proteins expression are needed to further clarify the role of SOCS2 in the immune response and on production traits in our *Socs2*-deficient sheep model.

To our knowledge, this is the first report of a mutation with pleiotropic effect in which a key molecule is identified as a potential tradeoff between health and production traits in dairy ruminants. Similarly Fasquelle et al. [42], found a mutation in the MRC2 gene which accounts for the outbreak of the Crooked Tail Syndrome in Belgian Blue Cattle while associated with enhanced muscular development in the general population. Further, Kadri et al. [43] showed that a 600-b deletion accounted for antagonistic effect on fertillity and milk yield in dairy Nordic Red Cattle. Both health and fertility deleterious variants were found at high frequency most probably because of selective advantage on production trait. The present results may, at least partly, explain the adverse genetic correlation between SCC and milk production that exists in our sheep population, i.e., 0.18 between SCS and milk yield in first lactation [13]. Such a genetic opposition has been widely documented in other dairy ruminant species [2,6,7,8,9] and corroborates the earliest genetic studies which provided evidence that the highly successful selection for milk production had probably led to a deterioration of mastitis resistance in cattle [44]. The genetic opposition between health and production caused by such genetic variants with pleiotropic effect can therefore not be disrupted by conventional- or SNP- assisted selection. We will therefore need to address the issue of how fast to minimize the frequency of the unfavorable *Socs2* genotype (if relevant) while minimizing the loss of genetic progress regarding production traits. The present finding and above mentioned cattle literature [42,43] add to the evidence that pleiotropy and tradeoffs are not uncommon in bred livestock. They highlight the need for knowledge and methods on how to achieve optimal balancing selection on both health and production traits in livestock, when specific variants with pleiotropic effect are identified.

### Conclusions

We have identified a mutation in the suppressor of cytokine signaling 2 (*Socs2*) gene that likely underlies a QTL for SCC in a dairy sheep model. This mutation is associated with persistently high milk cell counts, indicating chronic inflammation of the mammary gland.

The mutation modifies an amino acid (p.R96C) in the SH2 domain of SOCS2 and prevents binding to phosphorylated peptides from at least two endogenous receptors. Although the biochemical mechanisms by which SOCS2 alters the host’s response remain unknown, the fact that SOCS2 is known to play a key role in the negative feed-back of the cytokine-meditated response via JAK/STAT signalling pathways, supports the idea that our *Socs2*-deficient sheep fail to control the inflammatory process in response to intramammary bacterial infection, leading to impaired resistance to the disease.

The *Socs2* mutation was also associated with increased milk production, growth and size, which suggests a pleiotropic effect due to impairment of retro-control of the GH and prolactin signalling pathways. Altogether these results uphold further detailed analyses of the effect of SOCS2 on the immune response and production traits in our *Socs2*-deficient sheep model.

## Materials and Methods

### Discovery population and phenotypic measurements

For association mapping, data from a total of 1009 commercial French dairy rams were used. These 1009 males were distributed in 33 half-sib families in a so called grand-daughter design. Family size averaged 30.2 (±9.7) sons and ranged from to 18 to 54.

The phenotypic measurement was the milk somatic cell count (SCC) measured in lactating ewes and available from the national data base, (Centre de Traitement de l’Information Génétique, **CTIG**, Jouy en Josas, France), as well as pedigree information, as part of the official data system for livestock (ministerial order NOR: AGRT1431011A, 24^th^ March 2015, Ministry of Agriculture, France). Sampling methods and SCC analyses followed standard recommendations by the IDF (International Dairy Federation) and ICAR (International Committee for Animal Recording). SCC was measured on average three times per lactation in first and second parity. SCC was then log-transformed to somatic cell score [SCS = log2 (SCC/100) +3] to normalize the data distribution and averaged per lactation to compute the analyzed trait LSCS as described in [9]. For association mapping we used twice the daughter yield deviation (DYD) [45] for LSCS from the national genetic evaluation procedure [46]. DYDs correspond to the average performance of the daughters of a ram, corrected for the environmental effects and the genetic value of the dams.

### Genome-wide SNP genotyping

All 1009 rams were genotyped using the Illumina Ovine SNP50 BeadChip assay. DNA extraction from blood samples and genotyping were performed at the Laboratoire d’Analyses Génétiques pour les Espèces Animales, Jouy en Josas, France (LABOGENA; www.labogena.fr). Blood samples were collected as part of the official national preservation collection and the coded samples stored at LABOGENA. Data were cleaned using in-house pipelines automated for the whole dairy sheep population genotyping data. In brief, any individual with a call rate below 98% or showing pedigree inconsistency had been previously discarded (less than 3%). SNP quality control included the following inclusion criteria: call rate above 97%, minor allele frequency above 1% and Hardy-Weinberg P-value above 10^−6^. After edits, a total of 41,501 autosomal SNPs, distributed on ovine chromosomes OAR1 to OAR26 were included for further analyses. The marker order and positions were based on the Ovine Assembly v3.1 (http://www.livestockgenomics.csiro.au/sheep/oar3.1.php).

### Association mapping

For discovery association, both linkage analyses (LA) and genome-wide association study (GWAS) were applied to the data using the QTLMap software (Elsen et al., 1999; http://dga7.jouy.inra.fr/qtlmap/). For LA, interval mapping [47] was performed by likelihood ratio test (LRT) using within-sire linear regression [48]. The QTL effect (average substitution effect) was expressed in deviation units (SD) for the trait. GWAS was based on a regression analysis of the phenotypes on founder sires’ haplotypes for every haplotype of 4 consecutive SNPs along the chromosome [49]. Chromosome-wise significance levels were calculated with QTLMap, using the current family structure and phenotypes. For LA, the empirical 5% and 1% chromosome-wise significance levels of the test statistics were estimated from 1000 within-family permutations [50] for each chromosome. For GWAS, the empirical chromosome-wise significance level of the test statistics was estimated from 1000 simulations for each chromosome. The genome-wise thresholds were obtained by applying the Bonferroni correction: Pgenomewise = (1 – Pchromosomewise)^*n*^, where n is the number of chromosomes, i.e., 26 in sheep [51]. The 95% confidence intervals of the QTL locations were estimated by logarithm of odds drop-off [47] implemented in the QTLMap software. In practice, the bounds of the interval were the 2 locations where the likelihood was equal to the maximum likelihood minus 3.84 $(=\chi_{\left(1,0.05\right)}^{2})$.

### Sequencing and SNP calling

One paired-end library with a 300 bp insert size was generated for each animal using the Illumina TruSeq sample prep v2 Kit (PN FC-121-2001). 3 μg of each DNA were fragmented on a Covaris M220 focused-ultrasonicator and used to generate the libraries on a Tecan EVO200 automate. Quality controls were assessed with the High Sensitivity DNA chip (PN 5067–4626) on a 2100 Bioanalyzer (Agilent), and libraries were quantified using the Kappa Library Quantification kit (PN KK4824) on an ABI 7900 HT (Life Technologies). Each library was paired-end sequenced (2x100 bp) on a single lane of an Illumina HiSeq2000 flowcell using the Illumina TruSeq SBS Kit v3 (FC-401-3001).

FASTQ sequences were aligned to the sheep genome v3 (http://www.ensembl.org/Ovis_aries/Info/Index) with BWA software, version 0.7.0-r313 [52] (“aln” algorithm with default settings). The resulting SAM format files were processed using samtools view, sort and merge functions [52]. Then, we applied duplicate removal (http://picard.sourceforge.net, version 1.91), GATK (version 2.4.9) base quality score recalibration [53], indel realignment, and performed SNP and INDEL discovery and genotyping across all 3 samples simultaneously using standard hard filtering parameters. snpEFF (version 3.3a) and snpSIFT [54,55] were used to classify the filtered-by-genotype variations according to their functions as synonymous, nonsynonymous, nonsense, missense, insertions, deletions or splice variations. Results were further compared with the previously generated Illumina Ovine SNP50 BeadChip genotypes. Within the QTL localization interval, SNP satisfying the following characteristics were filtered: heterozygous in the father, homozygous (two different genotypes in the sons). Polymorphisms were visualized using IGV software [56].

### *Socs2* PCR genotyping test

Genotyping of the *Socs2* mutation was performed by allele-specific amplification using the KASPar SNP genotyping system, followed by fluorescence detection on an ABI7900HT. KASPar assays were carried out in 5 μL reactions according to the KBioscience published conditions (http://www.kbioscience.co.uk/). The primers used for this genotyping test are listed in S3 Table.

### SOCS2 cDNA preparation, cloning, sequencing and recombinant protein expression

Peripheral blood mononuclear cells (PBMC) from a *Socs2* heterozygous sheep were isolated from EDTA blood samples using centrifugation over Ficoll gradient, and cultured overnight in complete RPMI medium with 10% fetal calf serum. For cDNA preparation, a two-step purification of RNA was used with phenol-chloroform extraction followed by purification on silica columns (RNeasy mini kit, Qiagen). RNA integrity number (RIN) was determined using an Agilent 2100 bioanalyzer, and was above 6.5. cDNA was synthesized on 1 μg RNA with Superscript III reverse transcriptase (Invitrogen) following the manufacturer’s recommendations.

WT and p.R96C SOCS2 cDNA were cloned by PCR using primers OAR_*Socs2*-5’ and OAR_*Socs2*-3. PCR products were cloned in pCR2.1 plasmid using Strataclone PCR (Stratagene) and Sanger sequencing was performed with universal primers.

Sequences of each variant were transferred into prokaryotic vectors downstream of the Thioredoxin and 6xHis-tag sequences. Upon expression in BL21 *E*. *coli* strain, recombinant proteins were purified using His-tag and dialyzed overnight against a large volume of Tris-HCl 50 mM NaCl 150 mM pH 9 for refolding. Protein concentration was then determined using the Bradford reaction and purity of the recombinant proteins was monitored by SDS/PAGE and Coomassie blue staining.

### Surface Plasmon Resonance assays

All binding and kinetics studies based on surface plasmon resonance (SPR) technology were performed on a BIAcore T200 optical biosensor instrument (GE Healthcare). Immobilization of biotinylated phosphopeptides (Proteogenix) was performed on a streptavidin-coated (SA) sensorchip in HBS-EP buffer (10 mM Hepes pH 7.4, 150 mM NaCl, 3 mM EDTA, 0.005% surfactant P20) (GE Healthcare). All immobilization steps were performed at a flow rate of 2 μl/min with final peptide concentrations of 2.5 μg/ml and 25 ng/ml for 350 RU or 75 RU immobilized peptides, respectively. A channel (Fc2) was used for immobilization of GHR peptide and a channel with an immobilized scramble peptide (Fc1) was used as a reference surface for non-specific binding measurements. Binding analyses were performed with solubilized purified SOCS2-WT and SOCS2-p.R96C proteins at different concentrations over the immobilized peptide surface at 25°C for 2 minutes at a flow rate of 30 μl/min.

A single-cycle kinetics (SCK) analysis to determine association, dissociation and affinity constants (ka, kd, and KD respectively) was carried out by injecting different protein concentrations (100 nM–8.1 μM). Binding parameters were obtained by fitting the overlaid sensorgrams with the 1:1 Langmuir binding model of the BIAevaluation software version 1.0.

### Association of *Socs2* genotype with LSCS and milk production traits

For the 468 rams from the discovery population that were genotyped with the KASPar test, data from the national data base CTIG were used to quantify the effect of the *Socs2* genotype on milk production traits. A single SNP test of association by analysis of variance (ANOVA) was performed using the mixed procedure of the statistical analysis system (SAS) programs (9.1) for the 468 genotyped animals. The dependent variables were DYD for LSCS and production traits (Milk Yield, Fat and Protein content) available from the national genetic evaluation procedure. The three possible *Socs2* genotypes were fitted as a fixed explanatory variable and a significance threshold of p<0.05 was selected. The varcomp procedure of SAS was used to fit the genotype effect as random and estimate the proportion of variance explained by the genotype. The sire was included as a random effect in both mixed and varcomp models.

### Phenotyping and analyzing *Socs2* sheep for body weight and size

A group of 18 female sheep with the three *Socs2* genotypes were bred in the INRA experimental unit of La Fage (Causse du Larzac, 43°54'54.52′′N; 3°05'38.11′′E, Aveyron, France). The 18 sheep were triplets of *Socs2*-C/C,-C/T and -T/T genotype, each triplet being sired from a common ram, except one (C/T and T/T from common sire and a non-related C/C match). The sheep were born in 2010, 2011 and 2012. Body weight was measured to the nearest 0.1 Kg at 12 time points during a 3 year period, from birth to third lambing. Number of records per ewe was 11 on average and varied from 7 to 12. Repeated body weight data within animal were analyzed with a linear model using the mixed procedure of SAS (SAS Institute, Cary, NC). Fixed effects were *Socs2*-genotype within time point, year of birth and triplet match, whereas the animal was treated as a random effect. Body size was measured in 17 out of the 18 sheep once in October 2013 to obtain fourteen measurement points of height, width and bone lengths as illustrated in S1 Fig. Morphometric data were analyzed with a linear model based on GLM procedure from SAS (SAS Institute, Cary, NC) including the fixed effects of genotype, age at measure (1, 2 or 3 years old) and triplet match. A significance threshold of p<0.05 was declared for both analyses of weight and size.

### Ethics statement

Commercial rams did not belong to any experimental design but were sampled by veterinarians and/or under veterinarian supervision for routine veterinary care and DNA collection. For the experimental animals (INRA, Domaine de La Fage), breeding conditions were similar to commercial sheep flocks. Blood collection and measurements followed procedures approved by the Regional Ethics Committee on Animal Experimentation, Languedoc-Roussillon (France), under the Agreement 752056/00.

## Supporting Information

S1 Table QTLs for the milk somatic cell count in a grand-daughter design of 1009 dairy sheep. QTL detection was based on both linkage (LA) and haplotype-based association (GWAS) analyses using the 50K OvineSNP50Beadchip (Illumina, San Diego, CA). The 26 ovine autosomes were analyzed. The 95% confidence intervals of the QTL locations were estimated by logarithm of odds drop-off method. The QTL effect (average substitution effect) is expressed in deviation units (SD) for the trait. Significance thresholds: 5% (*) and 1% (**) chromosome wide (*) and 5% genome wide (***).
(DOCX)

S2 Table 207 candidate SNPs determined by whole-genome sequencing in the OAR3-QTL region. SNP were obtained in a trio of rams (father Qq, resistant son qq and susceptible son QQ) after quality and genotype filtering. For each variation, the position on sheep genome v3.0, the dbSNP ssnumber or dbVar reference, the reference base, the alternate sequence, quality index as determined by GATK, GT:AD:GQ:PL values for susceptible son, resistant son and father and annotation as determined by SNPEff are indicated. GT:AD:GQ:PL are GATK SNP calling results and indicate respectively the genotype (0 for reference base and 1 for alternate sequence), Allelic Depth for the reference and the alternate alleles, the genotype quality and the List of Phred-scaled genotype likelihoods for the 0/0, 0/1 and 1/1 genotypes.
(ODS)

S3 Table List of primers used in the study. For each primer, the DNA sequence (5’3’), strand, Location (bp) and Comments are indicated. Location of primers are based on the OARv3.1 assembly available on http://www.ensembl.org/Ovis_aries/Info/Index.
(DOCX)

S1 Fig Morphometric measurement collected in eighteen sheep. M1: Thoracic circumference (at elbow), M2: Thoracic circumference (at hypochondria), M3: Height at wither, M4: Height at sacrum, M5: Height at hock, M6: Height at elbow, M7: Breast width, M8: Width between elbows, M9: Width between ischium, M10: Width between hips, M11: Body length (from base of neck to base of tail), M12: Humerus length, M13: Femur length, M14: Tibia length.
(TIF)

S1 File Raw data for Fig 5 and Fig 6. (XLSM)
