# Supplementary material for: A Point Mutation in Suppressor of Cytokine Signalling 2 (Socs2) Increases the Susceptibility to Inflammation of the Mammary Gland while Associated with Higher Body Weight and Size and Higher Milk Production in a Sheep Model
Source: PLoS Genet. 2015 Dec 11;11(12):e1005629. doi: 10.1371/journal.pgen.1005629 (PMC4676722; doi:10.1371/journal.pgen.1005629)
Supplement: S1 Table — QTL detection was based on both linkage (LA) and haplotype-based association (GWAS) analyses using the 50K OvineSNP50Beadchip (Illumina, San Diego, CA). The 26 ovine autosomes were analyzed. The 95% confidence intervals of the QTL locations were estimated by logarithm of odds drop-off method. The QTL effect (average substitution effect) is expressed in deviation units (SD) for the trait. Significance thresholds: 5% (*) and 1% (**) chromosome wide (*) and 5% genome wide (***). (DOCX) [file pgen.1005629.s001.docx]

**Table S1. QTLs for the milk somatic cell count in a grand-daughter design of 1009 dairy sheep**

| **Chromosome** | **Significance Threshold** | **Method** | **Position**  **(Mb)** | **95% Confidence Interval** | **Substitution Effect** |
| --- | --- | --- | --- | --- | --- |
|  | * | GWAS | 22.65 | 22.6-22.8 |  |
| 3 | * | LA | 130.10 | 129.4-131.4 | 0.53 |
|  | *** | GWAS | 129.94 | 129.8-130.0 |  |
| 4 | *** | GWAS | 15.86 | 15.8-16.0 |  |
| 5 | * | GWAS | 102.69 | 102.6-102.8 |  |
| 6 | * | GWAS | 71.31 | 71.1-71.5 |  |
| 7 | ** | GWAS | 24.53 | 24.4-24.6 |  |
| 8 | * | LA | 82.60 | 81.4-83.5 | 0.47 |
|  | ** | GWAS | 59.85 | 59.7-59.9 |  |
| 9 | ** | GWAS | 27.49 | 27.4-27.6 |  |
| 10 | ** | LA | 49.30 | 48.9-49.6 | 0.52 |
|  | ** | GWAS | 74.84 | 74.7-74.9 |  |
| 11 | *** | LA | 36.70 | 35.8-41.1 | 0.60 |
|  | *** | GWAS | 41.18 | 41.0-41.3 |  |
| 12 | ** | GWAS | 13.84 | 13.7-13.9 |  |
| 13 | ** | GWAS | 70.89 | 70.8-71.1 |  |
| 14 | * | LA | 39.40 | 36.4-40.2 | 0.45 |
|  | ** | GWAS | 56.58 | 56.5-56.7 |  |
| 16 | *** | LA | 36.10 | 35.2-37.0 | 0.53 |
|  | ** | GWAS | 5.89 | 5.8-6.0 |  |
| 17 | * | GWAS | 23.65 | 23.5-23.7 |  |
| 18 | ** | GWAS | 31.18 | 30.9-31.3 |  |
| 19 | ** | GWAS | 28.60 | 28.4-28.7 |  |
| 20 | * | GWAS | 48.73 | 48.6-48.8 |  |
| 22 | * | GWAS | 48.47 | 48.4-48.6 |  |
| 23 | *** | GWAS | 59.94 | 59.8-60.0 |  |
| 24 | ** | GWAS | 73.00 | 7.2-7.4 |  |
| 26 | ** | GWAS | 19.57 | 19.5-19.7 |  |

QTL detection was based on both linkage (LA) and haplotype-based association (GWAS) analyses using the 50K OvineSNP50Beadchip (Illumina, San Diego, CA). The 26 ovine autosomes were analysed. The 95% confidence intervals of the QTL locations were estimated by logarithm of odds drop-off method. The QTL effect (average substitution effect) is expressed in deviation units (SD) for the trait. Significance thresholds: 5% (*) and 1% (**) chromosome wide (*) and 5% genome wide (***).
