# Supplementary material for: A Point Mutation in Suppressor of Cytokine Signalling 2 (Socs2) Increases the Susceptibility to Inflammation of the Mammary Gland while Associated with Higher Body Weight and Size and Higher Milk Production in a Sheep Model
Source: PLoS Genet. 2015 Dec 11;11(12):e1005629. doi: 10.1371/journal.pgen.1005629 (PMC4676722; doi:10.1371/journal.pgen.1005629)
Supplement: S3 Table — For each primer, the DNA sequence (5’3’), strand, Location (bp) and Comments are indicated. Location of primers are based on the OARv3.1 assembly available on http://www.ensembl.org/Ovis_aries/Info/Index. (DOCX) [file pgen.1005629.s003.docx]

| **Primers** | **Sequence (5 ’to 3’)** | **Strand** | **Location (bp)** | **Comments** |
| --- | --- | --- | --- | --- |
| KASP-SOCS2-up1 | CAGCTGGACCAACTAATCTT | plus | 129722180-129722199 | KASPAR genotyping of R96C SOCS2 SNP- wild-type sequence |
| KASP-SOCS2-up1bis | CAGCTGGACCAACTAATCTC | plus | 129722180-(129722199) | KASPAR genotyping of R96C SOCS2 SNP – mutated sequence |
| KASP-SOCS2-down1 | GTCCGCTTATCCTTGCACAT | minus | 129722308-129722327 | KASPAR genotyping of R96C SOCS2 SNP |
|  |  |  |  |  |
| OAR_SOCS2-5’ | ctgtctttgccgcggtga | plus | 129720488-129720505 | SOCS2 cDNA cloning |
| OAR_SOCS2-3’ | tctcttttacatagctgcattcgg | minus | 129722553- 129722576 |  |
|  |  |  |  |  |
| OAR_UBE2N | TACTTTAGGGGCTGCCATTG | plus | 129579715-129579734 | qPCR |
| OAR_UBE2N | CGGCATTAAAGCAGAACCAG | minus | 129579840-129579859 |  |
| OAR_MRPL42 | GGCCAGATCCAGTGCATAAC | plus | 129646321-129646342 |  |
| OAR_MRPL42 | GTTCTATCATGGGTCCTTGCTC | minus | 129646241-129646260 |  |
| OAR_SOCS2 | AAAGAGGCACCAGAAGGAAC | plus | 129722200-129722220 |  |
| OAR_SOCS2 | CCCATCTTGGTATTCGATGC | minus | 129720702-129720721 |  |
| OAR_CRADD | CCTCCGGAAAACAATGCTAC | plus | 129820466-129820485 |  |
| OAR_CRADD | TGCAGGGAATCTAGGAATGC | minus | 129820527-129820546 |  |
| OAR_PLXNC1 | GGCTGGAAGAAGCTCAGAAAC | plus | 130424348-130424369 |  |
| OAR_PLXNC1 | TCCACTTGCATTTCTTCTTTTC | minus | 130424298-130424318 |  |
|  |  |  |  |  |
