# Supplementary figures and images for: A Point Mutation in Suppressor of Cytokine Signalling 2 (Socs2) Increases the Susceptibility to Inflammation of the Mammary Gland while Associated with Higher Body Weight and Size and Higher Milk Production in a Sheep Model
Source: PLoS Genet. 2015 Dec 11;11(12):e1005629. doi: 10.1371/journal.pgen.1005629 (PMC4676722; doi:10.1371/journal.pgen.1005629)

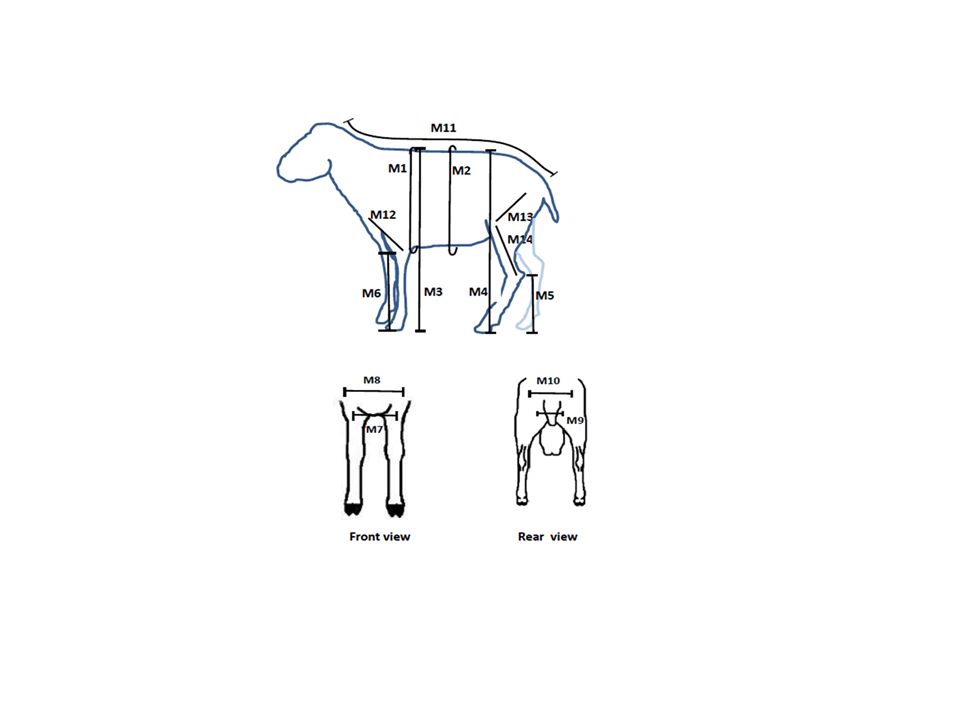

Supplement: S1 Fig — M1: Thoracic circumference (at elbow), M2: Thoracic circumference (at hypochondria), M3: Height at wither, M4: Height at sacrum, M5: Height at hock, M6: Height at elbow, M7: Breast width, M8: Width between elbows, M9: Width between ischium, M10: Width between hips, M11: Body length (from base of neck to base of tail), M12: Humerus length, M13: Femur length, M14: Tibia length. (TIF) [file pgen.1005629.s004.tif]
